# Metabolic and hormonal adaptation in *Bubalus bubalis* around calving and early lactation

**DOI:** 10.1371/journal.pone.0193803

**Published:** 2018-04-04

**Authors:** Enrico Fiore, Francesca Arfuso, Matteo Gianesella, Domenico Vecchio, Massimo Morgante, Elisa Mazzotta, Tamara Badon, Pasquale Rossi, Silvia Bedin, Giuseppe Piccione

**Affiliations:** 1 Department of Animal Medicine, Productions and Health (MAPS), University of Padua, Padua, Italy; 2 Department of Veterinary Sciences, University of Messina, Polo Universitario dell’Annunziata, Messina, Italy; 3 National Reference Centre on Water Buffalo Farming and Productions Hygiene and Technologies, Istituto Zooprofilattico Sperimentale del Mezzogiorno, Salerno, Italy; 4 Sud Rienergy, Corigliano Calabro, Italy; University of Illinois, UNITED STATES

## Abstract

Pregnancy and lactation are physiological periods that result in an increased metabolic demand that, if not satisfied, could provoke a threat to homeostasis. In this study changes in the values of Non-Esterified Fatty Acids (NEFA) and β-hydroxybutyrate (BHB), glucose, insulin, thyroid hormones, milk composition and yield were investigated in buffaloes during the late pregnancy and early lactation. From a total of 50 buffaloes, blood samples were collected -7±5 days before expected calving; +7±5; +30±5 and +50±5 days *post-partum*; milk samples were collected at the same *post-partum* time points. On serum samples, the values of Non-Esterified Fatty Acids (NEFA), β-hydroxybutyrate (BHB), glucose, insulin, Triiodothyronine (T3), Thyroxine (T4) and Thyroid-stimulating hormone (TSH) were evaluated. On milk, fat %, protein %, lactose %, somatic cells score (SCS), milk yield and daily milk production (DMP) were assessed. Peripartum period significantly influenced all studied parameters (P<0.05). Milk constituents and productivity statistically changed throughout monitoring period (P<0.005). Milk yield resulted positively correlated with insulin and TSH values, negatively correlated with NEFA and BHB. Insulin was negatively correlated with lactose % and positively correlated with SCS. The obtained results showed that the peripartum period and lactation are accompanied by marked changes in some biochemical variables and in the thyroid hormones values in Italian Mediterranean Buffaloes. Moreover, the relationship found between TSH values and milk yield seems to suggest a possible role of thyroid gland on the maintenance of lactogenesis. This study underlines the importance of monitoring the hormonal status of buffalo during the transition period in order to understand when adjustments of regulatory mechanisms break through physiological limits predisposing the buffalo to metabolic problems.

## Introduction

Immediately before parturition as well as during the first stage of lactation, increased mammary gland activity results in energy deficiency and increased lipomobilization from body reserves [1].

Despite the action of homeostatic mechanisms to maintain blood parameters within physiologic levels, changes in metabolites and hormones occur as a result of increased metabolic demands during both pregnancy and lactation [1–2]. These changes are not necessarily indicative of diseases but make pregnant animals physiologically unstable and more susceptible to a number of metabolic diseases at this stage than during other life periods compromising productivity [3]. Homeostasis control involves maintenance of physiological equilibrium or constancy of environmental conditions within the animal. Homeostasis is the orchestrated or coordinated control in metabolism of body tissues necessary to support a physiological state [4]. Peripartum period represents a critical life phase in buffaloes as well [5–7], since have to adjust metabolically to the increase in energy and nutrient requirements needed to ensure milk production [4–6].

Many authors investigated the buffalo metabolic response to lactation [5–6,8–10] since buffaloes show a different pattern compared to other ruminants as demonstrated by the low incidence of metabolic disorders [11]. Throughout the large literature on buffalo it is very difficult to find some problems of ketosis, and in a study the β-hydroxybutyrate (BHB), decrease after calving [8] or remain at low level [11]. However, some authors stated that, ketosis is still one of the major diseases that lead to a decrease the milk production during lactation in buffaloes [12].

Through the complex system of surveillance of metabolism it is possible to detect early aberrations in metabolic pathways and, with appropriate instructions, to correct ongoing disturbances [10,11,12]. The concentration of blood metabolites including Non-Esterified Fatty Acids (NEFA) and β-hydroxybutyrate (BHB), glucose and insulin may provide some indication of metabolic status and can be useful as a herd monitoring tool [13–14]. It is well stated that, adaptation of the endocrine system during the transition period is the key factor in maintaining metabolic balance. The centre of animal physiology is the homeostasis of the glucose, which involves primarily the somatotropine, insulin, glucagon and glucocorticoids with thyroid hormones being usually decreased after calving, with the aim to reduce tissue metabolism in order to have a higher nutrient availability for mammary gland metabolism [15]. Thyroid gland hormonal activity has an important role in the peripartum period for determining the cell metabolism intensity, the metabolism of lipids and carbohydrates and lactation course, as well [16]. Thyroid hormones modulate metabolism in ruminants in which carbohydrates and lipids are the major constituents [16].

In view of such considerations, the aim of the study was to evaluate the dynamic changes in the values of NEFA, BHB, glucose, insulin, TSH and thyroid hormones (T3, T4) in buffaloes during the late pregnancy and early lactation. We also aimed to evaluate the relationship of the considered blood metabolites and thyroid hormones with milk yield and composition.

## Material and methods

### Ethics statement

The protocol of this study was carried out according to the standards recommended by the Guide for the Care and Use of Laboratory Animals and Directive 2010/63/EU.

The study was concurrently performed with the competent authority and the authorized veterinarian during the official sanitary routine inspection to the farm. In Italy, the sanitary routine inspection of farm, including sampling of biological samples as blood or milk, don’t require an authorization, or an ID, or protocol number.

### Farm conditions and animals

A total of 50 multiparous Italian Mediterranean Buffaloes (*Bubalus bubalis*) with 114.5 ± 8.00 months old and 540 ± 55kg average body weight were randomly selected from a farm of 850 animals located in the Southern Italy (Corigliano Calabro, 39°36' N 16°31' E, 0 m.a.s.l, Cosenza). The average of milk production in 270 DIM of the buffaloes found in farm was 2827.45±654.60 kg per year with an average of 8.47% of milk-fat and 4.74% of milk protein.

All buffaloes had a dry period of 120 days in the farm. Total Mixed Ration (TMR) and the chemical composition of diets used during dry period and subsequent early lactation period is reported in Table 1. The composition of TMR was determined using near infrared reflectance spectroscopy (FOSS, NIRS^™^ DS2500). Water was available *ad libitum*.

**Table 1:** Feed chemical composition of Total Mixed Ratio (TMR) used for all animals during *pre-partum* and *post-partum* period.

	Dry Period	Lactation Period
Feed basis (kg)	19.20	23.20
Dry Matter (DM) (kg)	9.64	17.66
Dry Matter Intake (DMI) (kg per animal)	9.04	17.40
Chemical Composition (% of DM)		
Energy (UFL)	0.63	0.94
CP	9.12	15.40
PD	/	9.40
PDIN	6.08	1.55
PDIE	6.81	9.76
PDIA	2.51	4.83
UIP%DM	25.74	22.21
NDF	61.51	34.83
ADF	38.35	21.60
ADL	9.20	3.85
EE	2.61	5.44
ASH	8.52	7.22
ST	10.60	22.16
NSC	16.91	34.88
Ca	0.49	0.80
P	0.58	0.40

UFL: Unitè Fouragère Lait; CP: Crude protein; PD: protein digestible; PDIN: protein digested in the small intestine when rumen-fermentable nitrogen is limiting; PDIE: protein digested in the small intestine when rumen-fermentable energy is limiting; PDIA: dietary protein undegraded in the rumen but truly digestible in the small intestine; UIP%DM: percentage of undegradable intake protein on dry matter; NDF: neutral detergent fiber; ADF: acid detergent fiber; ADL: acid detergent lignin; EE: ether extract; ASH: Ashes; ST: Starch; NSC: non-structural carbohydrates; Ca: calcium; P: phosphorus

The farm was visited once a week with the authorized veterinarian during the official sanitary routine inspection to the farm and animals in proper stage were checked for their health status and they were sampled.

Their health status was evaluated on the basis of rectal temperature, heart rate, respiratory profile, appetite and fecal consistency. Twelve animals presented clinically manifested disease in *postpartum* period (four retain placenta, three uterine prolapses, two metritis, two mastitis, one milk fever); they were excluded from the study and and the data obtained from these animals were not considered for the subsequent statistical analysis. Therefore only the animals considered clinically healthy were enrolled in the study. Each animal was kept under natural photoperiod during winter season. The environmental temperature was 14±6°C.

According to Edmonson et al. [17], Body Condition Score (BCS) was evaluated on a scale from 0 to 5 during *pre-partum* period (at -7±5 days before calving) and *post-partum* period (+7±5; +30±5; +50±5days after calving).

### Blood sampling and chemistry analysis

From each animal blood sampling was performed at 4 different time points: -7±5 days before expected calving (-7±5 days *pre-partum*); +7±5 days; +30±5 days and +50±5 days after calving (+7±5 days *post-partum*; +30±5 days *post-partum*; +50±5 days *post-partum*). Blood sampling was performed early in the morning, before daily delivery of ration, by jugular venipuncture into10 mL vacuum tubes without anticoagulant agent (BD Vacutainer Systems, Preanalytical Solutions, Plymouth, UK).

Serum separation was carried out on field. The blood samples were allowed to clot for 30 min after blood sampling; afterwards the tubes were centrifuged at 1780g for 10 minutes. The obtained serum was transported to the laboratory at 4°C and then stored at -18°C until analysis. On serum samples, the concentration of Non-Esterified Fatty Acids (NEFA), β-hydroxybutyrate (BHB), glucose, insulin, Triiodothyronine (T3), Thyroxine (T4) and Thyroid-stimulating hormone (TSH) was assessed.

NEFA, BHB and glucose analysis were performed by BT1500 (Biotecnica Instruments S.p.a., Rome, Italy): NEFA concentration was determined by a colorimetric method, NEFA RX Monza test (kit no. FA 115, Randox, Crumlin, UK); BHB concentration was determined by RANBUT RX Monza test (kit no. RB 1007, Randox, Crumlin, UK); Glucose concentration was determined by Glucose Monoreagent, LR (Gesan S.r.l, Campobello di Mazara, Italy). Insulin concentration was quantified with a commercial IMMULITE^®^ 1000 kit (Commercial Kit INSULIN Siemens, Italy).

T3, T4, TSH concentrations in each sample were quantified with a commercial IMMULITE^®^ 1000 kit (Siemens, Italy): IMMULITE 1000 TOTAL T3; IMMULITE 1000 TOTAL T4; IMMULITE 1000 THIRD GENERATION TSH (TSH Reagent Wedge (LTS2), 7.5 mL alkaline phosphatse (bovine calf intestine) conjugated to polyclonal goat anti-TSH).

### Milk sampling and analysis

Daily milk production (DMP) was measured by means of a commercial milk meter Afimilk MCP (Afifarm). In addition, the milk production in the considered *postpartum* time points (at +7, at +30 and at +50 days *post-partum*) was calculated and named milk yield. For each animal two milk samples were manually collected from each daily milking, one for the determination of Somatic Cell Count (SCC) and the other one for milk composition, at 3 different after calving time points: +7±5, +30±5 and +50±5 days *post-partum*. Collection of mammary secretion was performed aseptically according to National Mastitis Council guidelines [18]. The teat ends were cleaned externally first with commercial pre-milking disinfectant solutions then dried with individual towel and cleaned again with alcohol.

The first few streams of foremilk were rejected and a pool of 25 mL of milk from the quarters was collected in sterile containers and preserved with sodium azide for assessing SCC with a Fossomatic cell counter (Foss Electric, Hillerod, Denmark). Milk samples were stored at 2–6°C and cultured until 24 hours. The SCC obtained values were converted to SCS (Somatic Cell Score) by:

SCS=3+log2(SCC÷100)

where SCC were in units of cells per microliter [19].

For the determination of milk composition, milk samples were analyzed using a lacto-scan (Milkotronic Analyzer, MCC, Nova Zagora, Bulgaria). The Net Energy Lactation(NE_L_) represents the energy contained in the milk produced. The NE_L_ concentration in milk is equivalent to the sum of heats of combustion of individual milk component (fat %, protein % and lactose %). Reported heats of combustion of milk fat, protein and lactose are 9.29, 5.71 and 3.95 Mcal/kg, respectively. NE_L_ concentration (Mcal/kg) in milk was calculated by the equation proposed by Tyrrell and Reid [20]:

$$NE_{L}=0.0929\times \left(Fat+0.0571\right)\times \left(Crude\;protein+0.0395\right)\times Lactose$$

### Statistical analysis

Data, expressed as mean values ± standard deviation (±SD), were tested for normality using the Shapiro-Wilk normality test. All data were normally distributed (P>0.05) and the statistical analysis was performed. One-way analysis of variance (ANOVA) for repeated measures was used to determine a statistically significant effect of peripartum period on haematochemical parameters, and in order to verify the effect of calving distance on productive parameters and milk constituents. Bonferroni’s multiple comparison test was applied for post-hoc comparison. The Person test was performed in order to assess significant correlations between haematochemical parameters and productive parameters and/or milk constituents. P value <0.05 was considered statistically significant. Statistical analysis was performed using the STATISTICA 7 software package (Stat Software Inc., Tulsa, Oklahoma, USA).

## Results

**Fig 1:**
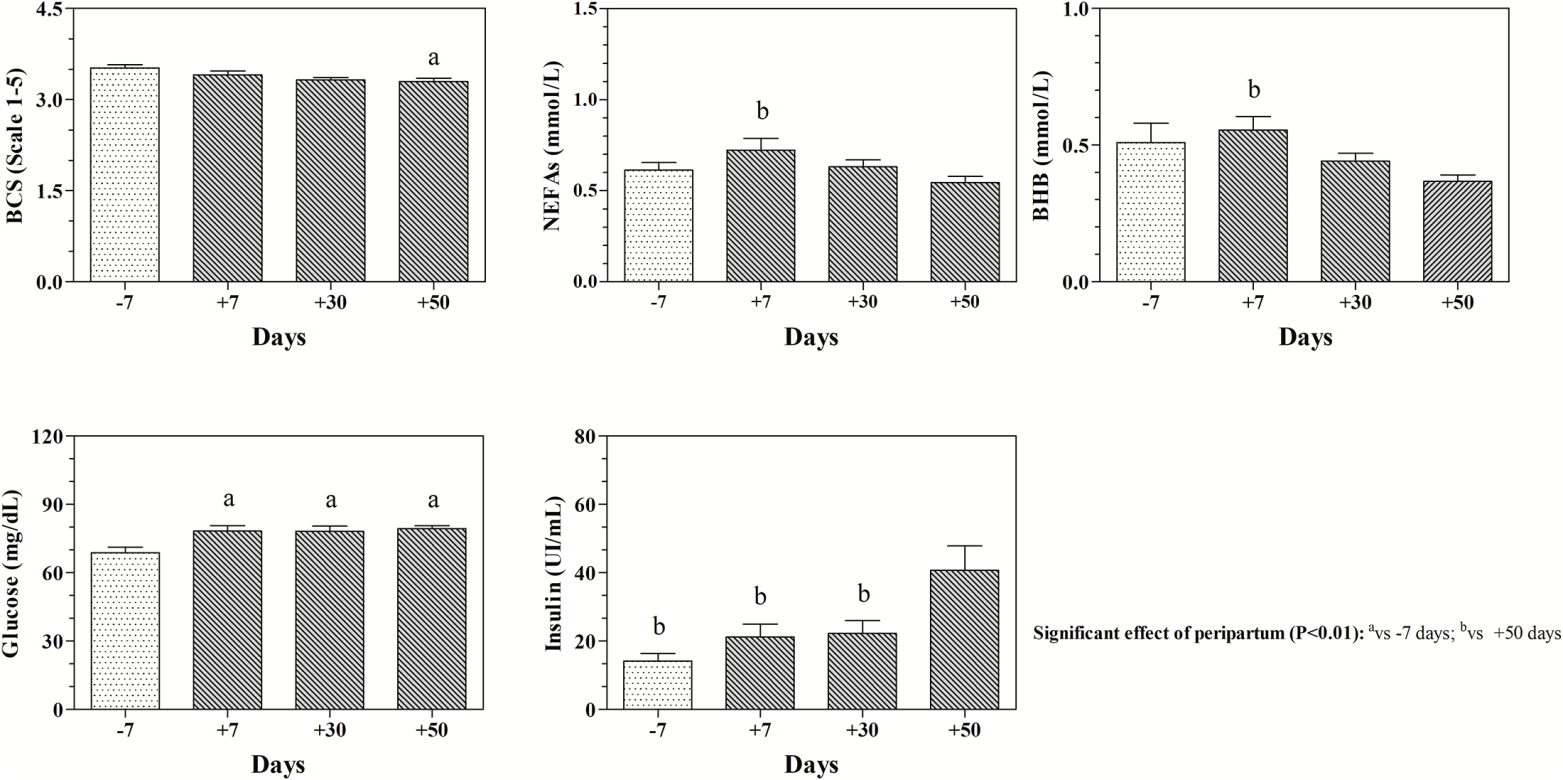
Means values (±SD) of Body Condition Score (BCS), Non Esterified Fatty Acids (NEFA), β-Hydroxuybutyrrate (BHB), glucose and insulin measured in buffaloes during *pre-partum* (-7 days) and *post-partum* (+7, +30 and +50 days) periods.

Statistical analysis showed a significant effect of peripartum period on the values of BCS (P<0.05), NEFA (P<0.05), BHB (P<0.05), glucose (P<0.01), insulin (P<0.01), T3 (P<0.01), T4 (P<0.01) and TSH (P<0.01). In particular, as showed in Fig 1, lower BCS values were found at 50 days *post-partum* respect to 7 days *pre-partum*; higher NEFA and BHB levels were found at 7 days *post-partum* respect to 50 days *post-partum*; lower glucose values were found at 7 days *pre-partum* respect to 7, 30 and 50 days *post-partum*, whereas insulin showed higher values at 50 days *post-partum* in comparison to 7 days *pre-partum*, 7days *post-partum* and 30 days *post-partum*.

**Fig 2:**
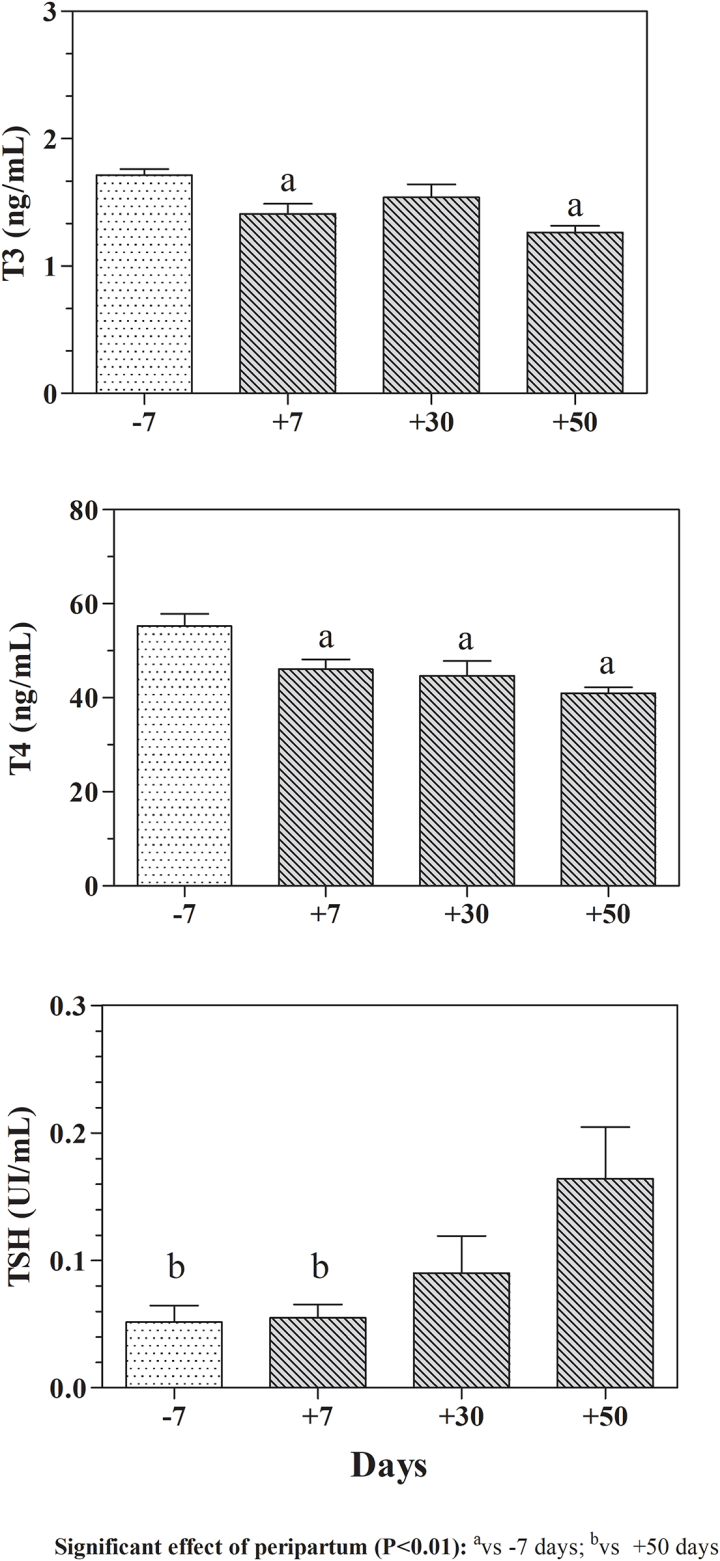
Means values (±SD) of Triiodothyronine (T3), Thyroxine (T4) and Thyroid-stimulating hormone (TSH) measured in buffaloes during *pre-partum* (-7 days) and *post-partum* (+7, +30 and +50 days) periods.

Fig 2 showed the trend of thyroid hormones values measured throughout the monitoring period. A gradual decrease in T3 and T4 values was found throughout the *post-partum* period, whereas THS showed an increasing trend with the highest values at 50 days *post-partum*.

**Fig 3:**
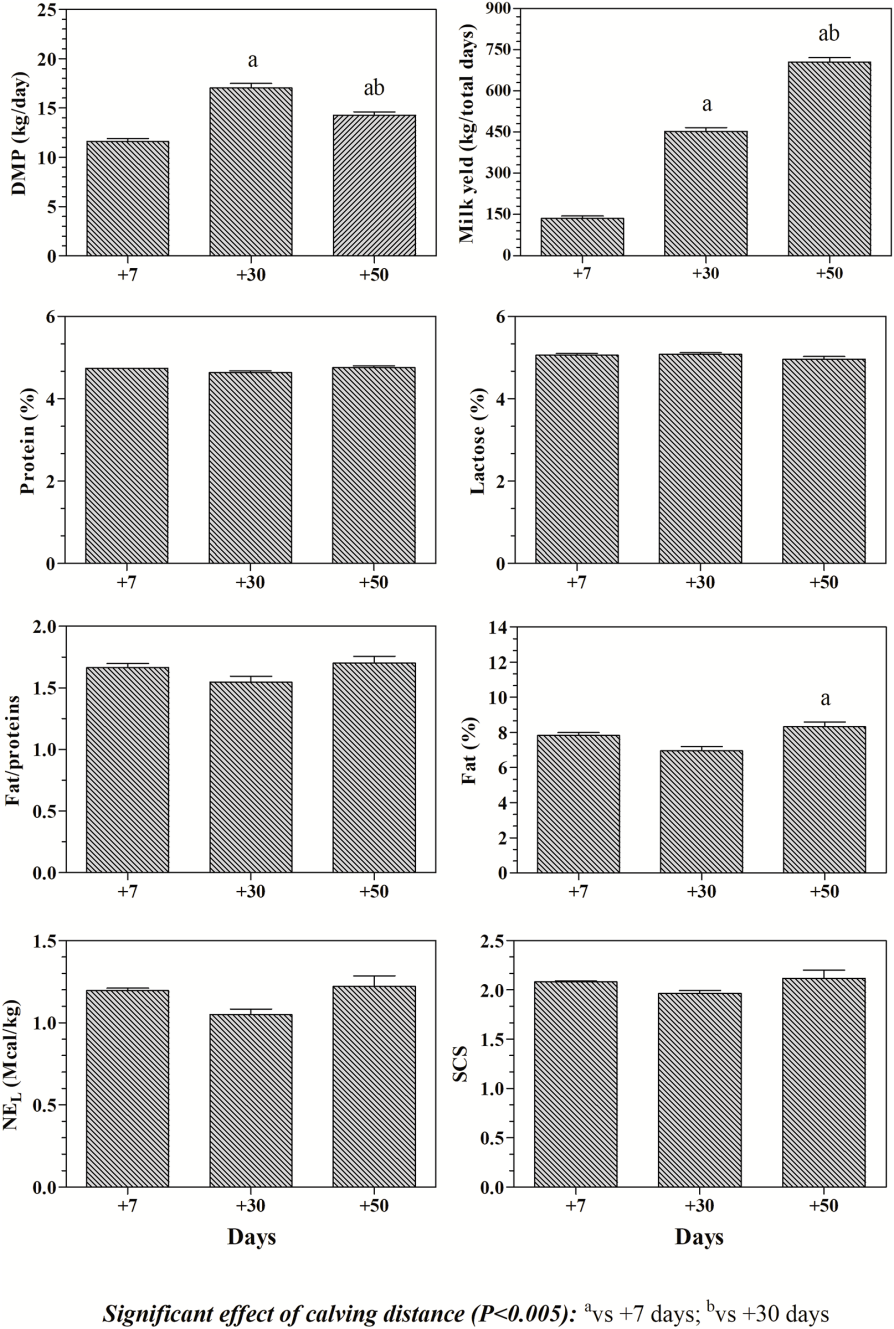
Means values (±SD) of productive parameters (milk yield; daily milk production, DMP; Net Energy Lactation, NE_L_) and milk constituents (fat %; lactose %; protein %; fat/protein; somatic cells score, SCS) according to calving distance.

Statistical effect of calving distance (P<0.005) was found on productive parameters and milk constituents (Fig 3). Milk yield showed an increasing trend throughout the *post-partum* period with the highest values at 50 days *post-partum*. DMP showed the lowest values at 7days *post-partum*. Fat % was significantly lower at 30 days *post-partum* respect to 50 days *post-partum*.

Pearson’s correlation results are reported in Table 2. The values of BHB and NEFA were negatively correlated with milk yield. A positive correlation was found between insulin and milk yield and SCS values, whereas it was negatively correlated with lactose % values. DMP resulted negatively correlated with BHB, NEFA and milk fat%. Milk lactose % was negatively correlated with SCS, milk fat % and milk protein %. The values of milk fat % were negatively correlated with NEFA, and positively correlated with milk protein %. A positive correlation was found between TSH values and milk yield.

**Table 2:** Significant Pearson’s correlation results found between milk and the other studied parameters. P values <0.05 were considered statistically significant.

	NEFA	BHB	Insulin	Glucose	BCS	TSH	T3	T4
**Milk yield**	r = -0.34	r = -0.36	r = +0.34	r = -0.03	r = -0.16	r = +0.28	r = -0.17	r = -0.19
	P < 0.005	P < 0.005	P < 0.005	P = 0.15	P = 0.17	P < 0.05	P = 0.15	P = 0.10
**DMP**	r = -0.23	r = -0.12	r = +0.14	r = -0.19	r = -0.11	r = +0.08	r = -0.007	r = -0.07
	P = 0.06	P = 0.31	P = 0.25	P = 0.10	P = 0.35	P = 0.45	P = 0.95	P = 0.54
**Fat %**	r = -0.02	r = -0.14	r = -0.03	r = -0.007	r = -0.04	r = +0.22	r = -0.07	r = +0.10
	P = 0.80	P = 0.20	P = 0.77	P = 0.95	P = 0.68	P = 0.06	P = 0.55	P = 0.38
**Protein %**	r = -0.03	r = -0.06	r = +0.21	r = +0.16	r = -0.04	r = -0.14	r = +0.003	r = -0.18
	P = 0.73	P = 0.55	P = 0.07	P = 0.16	P = 0.74	P = 0.22	P = 0.98	P = 0.11
**Fat/protein**	r = +0.0007	r = -0.14	r = -0.15	r = -0.08	r = -0.03	r = +0.20	r = -0.04	r = +0.06
	P = 0.99	P = 0.20	P = 0.19	P = 0.49	P = 0.82	P = 0.07	P = 0.72	P = 0.56
**Lactose %**	r = +0.12	r = +0.14	r = -0.26	r = -0.06	r = -0.08	r = -0.02	r = +0.04	r = +0.11
	P = 0.29	P = 0.24	P < 0.05	P = 0.55	P = 0.51	P = 0.90	P = 0.74	P = 0.32
**NE**_**L**_	r = +0.08	r = -0.08	r = -0.16	r = -0.05	r = -0.04	r = +0.19	r = -0.07	r = +0.13
	P = 0.49	P = 0.45	P = 0.15	P = 0.65	P = 0.74	P = 0.10	P = 0.55	P = 0.27
**SCS**	r = -0.17	r = -0.02	r = -0.25	r = -0.06	r = -0.003	r = -0.01	r = -0.002	r = -0.08
	P = 0.13	P = 0.87	P < 0.05	P = 0.61	P = 0.98	P = 0.93	P = 0.98	P = 0.51

## Discussion

Buffalo milk is nearly twice richer in fat compared to cow milk. The results obtained in this study showed the highest DMP values and the lowest milk fat % at 30 d *post-partum* according to the findings of previous studies carried out on buffalo species [10, 21]. According to Bertoni et al. [22], in our study the protein % was stable during lactation with values similar to the ones reported by Rosati and Van Vleck [21]. In agreement with the findings of a previous study carried out on periparturient buffaloes [23], the results obtained in the present study showed lower BCS values at *post-partum* respect to *pre-partum* time. This is common in early lactation, probably due to the higher energy requirement for milk production and the consequent fat mobilization and also because dietary energy does not depend only by concentration but also on feed intake. NEFA and BHB levels showed opposite trends respect to BCS values. In particular, NEFA showed increased values 7 days *post-partum* suggesting that fat mobilization starts with the beginning of lactation in buffalo in order to satisfy the high energetic requirements of animals at the onset of lactation [24]. Afterwards, NEFA values showed a decreasing trend at 50 days *post-partum*. It is well known that the increase of NEFA concentration leads to the increase of ketogenesis by hepatocytes [1, 15, 24]. In addition, the increase of ketones also depends on the availability of glucose. In this study, no overproduction of BHB was recorded and the values obtained from each animal throughout the monitoring period were within the physiological range established for buffalo indicating an appropriate fat metabolism [25]. Although it has been stated that ketosis is still one of the major diseases that lead to a decrease the milk production during lactation in buffaloes [12], our results seems to suggest that peripartum buffaloes enrolled in the study did not susceptible to ketosis. This finding disagrees with the results of Youssef et al. [12] showing that ketosis is still one of the major diseases that lead to a decrease the milk production during lactation in buffaloes. The values of NEFA and BHB were negatively correlated with milk yield throughout *post-partum* period. Together, these findings seem to suggest that buffalo nutritive and energetic requirements were satisfied during the lactation phase. The serum glucose values showed lower levels at *pre-partum* period compared to *post-partum* time points suggesting a greater rate of gluconeogenesis after calving, sufficient to cover the lactose synthesis [26]. The values of glucose remained stable throughout the *post-partum* period. It has been showed that in the healthy dairy cows glucose is reduced only after calving and not before because the glucose needs is very low at the stage of pregnancy [15]. The steady pattern of serum glucose concentration found in this study in *post-partum* buffaloes may indicate lack of changes in the absolute rate of gluconeogenesis and glycogenolysis. As described by Bertoni et al. [11], glucose is the only parameter among those involved in energetic metabolism in buffaloes that is not affected by lactation; probably, this is due to an efficient glucose homeostasis between insulin-dependent and insulin-independent pathways during lactation. Lactation impairs both the capacity for lipogenesis in ruminant adipose tissue and the ability of insulin to stimulate glucose uptake by adipose tissue and skeletal muscle due to the lower sensitivity of adipose and muscle cells to insulin. Crucially for the partitioning of nutrient in ruminants and despite the presence of insulin receptors in the ruminant placenta and mammary gland, glucose uptake by these tissues is not responsive to insulin [27]. According to a previous research carried out on cows [28], in the present study the levels of insulin were low at *pre-partum* and showed a slight increase starting from 7 days *post-partum* reaching the highest values 50 days *post-partum*. Insulin secretion in ruminants is increased by feeding. In ruminant, the effects of nutrients on insulin occur much later. The insulin response to feeding is increased when animals are fed with a high-concentrate diet [27]; however, the effect of high concentrate diet is at long term and very small in the early lactation [27, 29]. In the present study buffaloes come from an intake in the dry period of 9 kg of DM to reach an intake of 17.4 kg of DM during lactation; this passage needs time as digestive system, and in particular rumen, must adapt. When this is completed often in buffaloes energy intake (NEL) is higher than NEL in milk, so insulin increase [29]. Insulin is recognized as essential for the maintenance of normal lactogenesis although its exact mammary gland action is difficult to distinguish from its general anabolic role [30]. In the present study, it has been found a positive correlation between the values of insulin and milk yield, and between insulin and SCS values; whereas insulin levels resulted negatively correlated with lactose %. The positive correlation found between insulin values and milk yield could be due to the increase of glucose availability at blood level. Increased glucose availability is likely related to higher milk yield as well as to higher insulin release.

Even the thyroid gland function has an important role in the transitional period for lactation course [31]. Our results confirm that the peripartum period is accompanied by marked changes in circulating thyroid hormone profile in Italian Mediterranean Buffaloes as demonstrated in cows [32]. In particular, higher T3 and T4 values were found at *pre-partum* compared to *post-partum* period. TSH showed an opposite trend, since it increase from 7 days *pre-partum* reaching the highest value at 50 day *post-partum*. Serum thyroid levels are, of course, commonly used as an indication of tissue thyroid activity. However, in order to have biological activity, T4 and T3 must cross the cellular membrane from the serum into the target cells. The TSH act on thyroid gland, but the level of T3 is also affected by peripheral conversion of T4 to T3 or to the reverse T3. Moreover, reduced T3 and T4 transport into the cells in peripheral tissues are shown in a wide range of common conditions, including insulin resistance and physiological stress. Physiological stress was associated with reduced tissue levels of T4 and T3 without a corresponding increase in TSH [33]. This could explain the higher serum levers of these hormones found around calving known as a stress event for the animals [1,2]. Moreover, the pituitary has different transporters of T3 and T4 respect to the other tissue in the body. The thyroid transporters in the body are very energy dependent and are affected by numerous conditions, including low energy states, stress conditions, whereas the pituitary remains unaffected and is able to maintain intracellular T3 levels and, consequently, there is no increase of TSH [33]. The values of TSH were positively correlated with milk yield suggesting a possible role of thyroid gland on the start of lactogenesis; however, the action of thyroid gland on lactogenesis it is still to be clarified and confirmed by further studies in this field. The decreasing trend of serum T3 and T4 values we found in *post-partum* period could reflect the reduced thyroid hormone secretion rate probably due to the increase of the number of receptors for these hormones in the mammary gland [34]. At the beginning of lactopoiesis, in fact, there is an increase in the number of T3 receptors in the mammary gland secretory cells during lactation [34]; moreover, there is an actual secretion of T4 through the milk, which may represent between 4% and 7% of the total required for the maintenance of metabolic functions [35]. In addition, the decrease in T4 concentrations could yet be caused by the additional mammary gland secretion of maternal iodine during lactation.

## Conclusion

The results obtained in the present study showed that the peripartum period and lactation are accompanied by changes in the levels of some metabolic parameters and of circulating thyroid hormones in Italian Mediterranean Buffaloes; however, the dairy buffaloes seem to be not predisposed to metabolic problems linked to energy metabolism. Since highest NEFA values were found at 7 days *post-partum* before the achievement of the peak of DMP that occurred 30 days *post-partum*, the increase of serum NEFA levels at critical points of the transition period could highlight the importance of this parameter as indicator of energy balance in buffaloes as well. However, since these changes are small respect to dairy cows, further studies on this field are needed to better understand the role of NEFA as indicator of energy balance. The relationship found between TSH values and milk yield seems to suggest a possible role, although still unclear, of thyroid gland on the maintenance of lactogenesis.

Our findings underline the importance of monitoring the hormonal status of buffalo during the transition period in order to understand when adjustments of regulatory mechanisms break through physiological limits predisposing the buffalo to metabolic problems.

## References

[1] Arfuso F, Fazio F, Levanti M, Rizzo M, Di Pietro S, Giudice E, et al Lipid and lipoprotein profile changes in dairy cows in response to late pregnancy and the early postpartum period. Archives Animal Breeding. 2016; 59: 429–34.

[2] Ashmawy NA. Changes in peripheral plasma hormone concentrations and metabolites during the last trimester of pregnancy and around parturition in the Egyptian buffalo and Baladi cows. International Journal of Advanced Research. 2015; 3: 1377–90.

[3] Ashmawy NA. Blood metabolic profile and certain hormones concentrations in Egyptian buffalo during different physiological states. Asian Journal of Animal and Veterinary Advances. 2015; 10 (6): 271–80.

[4] Bauman DE, Currie WB. Partitioning of nutrients during pregnancy and lactation: a review of mechanisms involving homeostasis and homeorhesis.Journal of Dairy Science. 1980; 63(9): 1514–29. 7000867 10.3168/jds.s0022-0302(80)83111-0

[5] Abdulkareem TA. Some hematological and blood biochemical profile of Iraqi riverine buffaloes (*Bubalus bubalis*) during different gestation period. Journal of Buffalo Science (India). 2013; 2: 78–84.

[6] Abdulkareem TA. Some hematological and blood biochemical attributes of Iraqi riverine buffaloes (*Bubalus bubalis*) around calving and postpartum periods. Al-Anbar Journal of Veterinary Science. 2013; 6: 143–50.

[7] Fiore E, Giambelluca S, Morgante M, Contiero B, Mazzotta E, Vecchio D, et al Changes in some blood parameters, milk composition and yield of buffaloes (*Bubalus bubalis*) during the transition period. Animal Science Journal. 2017; 88: 2025–32. doi: 10.1111/asj.12872 28776872 10.1111/asj.12872

[8] Campanile G, Di Palo R, D’Angelo A. Haematological profile in buffalo. In: Proceedings of the 3rd Course on Biotechnology of Reproduction in Buffaloes 6–10 October Caserta, Italy. 1997; 236–49.

[9] Abdulkareem TA, Al-Sharifi SAM, Eidan SM, Sasser RG. Productive and reproductive performance of Iraqi buffalo as influenced of pre-mating and pre-calving concentrate supplementation. Pakistan Veterinary Journal. 2012; 32: 345–48.

[10] Grasso F, Terzano GM, De Rosa G, Tripaldi C, Napolitano F. Influence of housing conditions and calving distance on blood metabolites in water buffalo cows. Italian Journal of Animal Science.2004; 3: 275–82.

[11] Bertoni G, Lombardelli R, Piccioli Cappelli F, Bartocci S, Amici A. Alcuni fattori che influenzano le condizioni endocrine–metaboliche della specie bufalina. Agricoltura e Ricerca. 1994; 16: 87–98.

[12] Youssef MA, El-Khodery SA, El-deeb WM, Abou El-Amaiem WE. Ketosis in buffalo (*Bubalus bubalis*): clinical findings and the associated oxidative stress level. Tropical Animal Health and Production. 2010; 42:1771–77. doi: 10.1007/s11250-010-9636-9 20582718 10.1007/s11250-010-9636-9

[13] Ospina PA, Nydam DV, Stokol T, Overton TR. Evaluation of nonesterified fatty acids and beta-hydroxybutyrate in transition dairy cattle in the northeastern United States: Critical thresholds for prediction of clinical diseases. Journal of Dairy Science. 2010; 93: 546–54. doi: 10.3168/jds.2009-2277 20105526 10.3168/jds.2009-2277

[14] Ospina PA, Nydam DV, Stokol T, Overton TR. Associations of elevated nonesterified fatty acids and β-hydroxybutyrate concentrations with early lactation reproductive performance and milk production in transition dairy cattle in the northeastern United States. Journal of Dairy Science. 2010; 93: 1596–603. 20338437 10.3168/jds.2009-2852

[15] Fiore E, Barberio A, Morgante M, Rizzo M, Giudice E, Piccione G, et al Glucose infusion response to some biochemical parameters in dairy cows during the transition period. Animal Science Papers and Reports. 2015; 33: 129–36.

[16] Huszenicza GY, Kulcsar M, Rudas P. Clinical endocrinology of thyroid gland function in ruminants. Veterinarni Medicina Czech. 2002;47: 199–210.

[17] Edmonson AJ, Lean IJ, Weaver LD, Farver T, Webster G.A body condition scoring chart for Holstein Dairy Cows. Journal of Dairy Science. 1989; 72: 68–78.

[18] Hogan JS, Gonzalez RN, Harmon RJ, Nickerson SC, Oliver SP, Pankey JW, et al Laboratory handbook on bovine mastitis, Rev. edn National Mastitis Council Inc., Madison, USA; 1999.

[19] Wiggans GR, Shook GE. Lactation measure of somatic cell count. Journal of Dairy Science. 1987; 70: 2666–72. doi: 10.3168/jds.S0022-0302(87)80337-5 3448115 10.3168/jds.S0022-0302(87)80337-5

[20] Tyrrell HF, Reid JT. Prediction of the energy value of cow’s milk. Journal of Dairy Science. 1965; 48: 1215–23. doi: 10.3168/jds.S0022-0302(65)88430-2 5843077 10.3168/jds.S0022-0302(65)88430-2

[21] Rosati A, Van Vleck LD. Estimation of genetic parameters for milk, fat, protein and mozzarella cheese production for the Italian river buffalo *Bubalus bubalis* population. Livestock Production Science. 2002; 74: 185–90.

[22] Bertoni G, Piccioli Cappelli F, Bernabucci U, Di Stefano E. The changes in milk composition and blood parameters during the lactation cycle in dairy buffaloes. In: Proceeding of the International Symposium Prospects of Buffalo Production in the Mediterranean and the Middle East 9–12 November Cairo, Egypt; 1993; 270–72.

[23] Deka RS, Veena M, Kumar M, Shiwajirao ZS, Tyagi AK, Harjit K. Body condition, energy balance and immune status of periparturient murrah buffaloes (*Bubalus bubalis*) supplemented with inorganic chromium. Biological Trace Element Research. 2014; 161: 57–68. doi: 10.1007/s12011-014-0069-6 25037066 10.1007/s12011-014-0069-6

[24] Fiore E, Piccione G, Perillo L, Barberio A, Manuali E, Morgante M, et al Hepatic lipidosis in high-yielding dairy cows during the transition period: haematochemical and histopathological findings. Animal Production Science. 2015; 57: 74–80.

[25] Nozad S, Ramin AG, Moghadam G, Asri-Rezaei S, Babapour A, Ramin S. Relationship between blood urea, protein, creatinine, triglycerides and macro-mineral concentrations with the quality and quantity of milk in dairy Holstein cows. Veterinary Research Forum. 2012; 3: 55–59. 25653747 PMC4312820

[26] Medhammar E, Wijesinha-Bettoni R, Stadlmayr B, Nilsson E, Charrondiere UR, Burlingame B. Composition of milk from minor dairy animals and buffalo breeds: a biodiversity perspective.Journal of the Science of Food and Agriculture. 2012; 92: 445–74. doi: 10.1002/jsfa.4690 22083874 10.1002/jsfa.4690

[27] Tsuda T, Sasaki Y, Kawashima R. Physiological aspects of digestion and metabolism in ruminants. San Diego, CA, USA:Academic Press Inc.; 1991.

[28] Accorsi PA, Govoni N, Gaiani R, Pezzi C, Seren E, Tamanini C. Leptin, GH, PRL, insulin and metabolic parameters throughout the dry period and lactation in dairy cows. Reproduction in Domestic Animals. 2005; 40: 217–23. doi: 10.1111/j.1439-0531.2005.00581.x 15943695 10.1111/j.1439-0531.2005.00581.x

[29] Campanile G, Di Palo R, Esposito L, Boni R, Di Meo C. Variazioni di alcune costanti ematiche in bufale in lattazione. In: Proceedings of the 11th National Congress A.S.P.A. 19–22 June, Grado (GO), Italia. 1995; 77–78.

[30] Ruckebusch Y, Phaneuf LP, Dunlop R. Physiology of small and large animals. Philadelphia, Hamilton, USA: B.C. Decker Inc.; 1991.

[31] Nikolic JA, Šamanc H, Begovic J, Damjanovic Z, Dokovic R, Kostic G, et al Low peripheral serum thyroid hormone status independently affects the hormone profiles of healthy and ketotic cows during the first week postpartum. Acta Veterinaria Belgrade. 1997; 47: 3–14.

[32] Pethes GY, Bokori J, Rudas P, Frenyó VL, Fekete S. Thyroxin, triiodothyronine, reverse-triiodothyronine and other physiological characteristics of periparturient cows fed restricted energy. Journal of Dairy Science. 1985; 68: 1148–54. doi: 10.3168/jds.S0022-0302(85)80941-3 3842853 10.3168/jds.S0022-0302(85)80941-3

[33] Holtorf K. Thyroid hormone transport into cellular tissue Journal of Restorative Medicine. 2014; 3: 53–68.

[34] Wilson DB, Gorewit RC. Specific thyroxine receptors in mammary cytosol from lactating cattle. Biochemical and Biophysical Research Communications. 1980; 95: 807–15. 6251820 10.1016/0006-291x(80)90859-1

[35] Akasha M, Anderson RR. Thyroxine and triiodothyronine in milk of cows, goats, sheep, and guinea pigs. Proceedings of the Society for Experimental Biology and Medicine. 1984; 177: 360–71. 6483867 10.3181/00379727-177-41957

